# Longitudinal monitoring of Apparent Diffusion Coefficient (ADC) in patients with prostate cancer undergoing MR-guided radiotherapy on an MR-Linac at 1.5 T: a prospective feasibility study

**DOI:** 10.2478/raon-2023-0020

**Published:** 2023-06-21

**Authors:** Haidara Almansour, Fritz Schick, Marcel Nachbar, Saif Afat, Victor Fritz, Daniela Thorwarth, Daniel Zips, Felix Bertram, Arndt-Christian Müller, Konstantin Nikolaou, Ahmed E Othman, Daniel Wegener

**Affiliations:** Department of Diagnostic and Interventional Radiology, Eberhard-Karls University, Tuebingen, Germany; Section for Experimental Radiology, Department of Radiology, Eberhard-Karls University, Tuebingen, Germany; Department of Radiation Oncology, Charité University Medicine Berlin, Berlin, Germany; Section for Biomedical Physics, Department of Radiation Oncology, Eberhard-Karls University, Tuebingen, Germany; German Cancer Consortium (DKTK), Partner Site Tuebingen and German Cancer Research Center (DKFZ), Heidelberg, Germany; Department of Radiation Oncology, Eberhard-Karls University, Tuebingen, Germany; Department of Radiation Oncology, RKH Klinikum Ludwigsburg, Ludwigsburg, Germany; Cluster of Excellence iFIT (EXC 2180) “Image Guided and Functionally Instructed Tumor Therapies”, University of Tuebingen, Tuebingen, Germany; Department of Neuroradiology, University Medical Center Mainz, Mainz, Germany

**Keywords:** prostate carcinoma, MRI, adaptive radiotherapy, image guidance, MR-Linac, ADC

## Abstract

**Background:**

Hybrid MRI linear accelerators (MR-Linac) might enable individualized online adaptation of radiotherapy using quantitative MRI sequences as diffusion-weighted imaging (DWI). The purpose of this study was to investigate the dynamics of lesion apparent diffusion coefficient (ADC) in patients with prostate cancer undergoing MR-guided radiation therapy (MRgRT) on a 1.5T MR-Linac. The ADC values at a diagnostic 3T MRI scanner were used as the reference standard.

**Patients and and methods:**

In this prospective single-center study, patients with biopsy-confirmed prostate cancer who underwent both an MRI exam at a 3T scanner (MRI_3T_) and an exam at a 1.5T MR-Linac (MRL) at baseline and during radiotherapy were included. Lesion ADC values were measured by a radiologist and a radiation oncologist on the slice with the largest lesion. ADC values were compared before *vs*. during radiotherapy (during the second week) on both systems via paired t-tests. Furthermore, Pearson correlation coefficient and inter-reader agreement were computed.

**Results:**

A total of nine male patients aged 67 ± 6 years [range 60 – 67 years] were included. In seven patients, the cancerous lesion was in the peripheral zone, and in two patients the lesion was in the transition zone. Inter-reader reliability regarding lesion ADC measurement was excellent with an intraclass correlation coefficient of (ICC) > 0.90 both at baseline and during radiotherapy. Thus, the results of the first reader will be reported. In both systems, there was a statistically significant elevation of lesion ADC during radiotherapy (mean MRL-ADC at baseline was 0.97 ± 0.18 × 10^−3^ mm^2^/s *vs*. mean MRL-ADC during radiotherapy 1.38 ± 0.3 × 10^−3^ mm^2^/s, yielding a mean lesion ADC elevation of 0.41 ± 0.20 × 10^−3^ mm^2^/s, p < 0.001). Mean MRI_3T_-ADC at baseline was 0.78 ± 0.165 × 10^−3^ mm^2^/s *vs*. mean MRI_3T_-ADC during radiotherapy 0.99 ± 0.175 × 10^−3^ mm^2^/s, yielding a mean lesion ADC elevation of 0.21 ± 0.96 × 10^−3^ mm^2^/s p < 0.001). The absolute ADC values from MRL were consistently significantly higher than those from MRI_3T_ at baseline and during radiotherapy (p < = 0.001). However, there was a strong positive correlation between MRL-ADC and MRI_3T_-ADC at baseline (*r* = 0.798, p = 0.01) and during radiotherapy (*r* = 0.863, p = 0.003).

**Conclusions:**

Lesion ADC as measured on MRL increased significantly during radiotherapy and ADC measurements of lesions on both systems showed similar dynamics. This indicates that lesion ADC as measured on the MRL may be used as a biomarker for evaluation of treatment response. In contrast, absolute ADC values as calculated by the algorithm of the manufacturer of the MRL showed systematic deviations from values obtained on a diagnostic 3T MRI system. These preliminary findings are promising but need large-scale validation. Once validated, lesion ADC on MRL might be used for real-time assessment of tumor response in patients with prostate cancer undergoing MR-guided radiation therapy.

## Introduction

Radiotherapy (RT) is a curative treatment option for patients with localized prostate cancer.^1^ MR-guided radiotherapy (MRgRT) enables improved soft tissue contrast and enhances accuracy of treatment planning.^2^ In this context, the hybrid magnetic resonance 1.5T scanner with a linear accelerator MR-Linac (MRL) is currently being used in centers around the world to perform high-precision MRgRT with daily plan adaptations based on anatomical MR sequences.^3,4^ Furthermore, functional MRI sequences such as diffusion weighted imaging (DWI) are being additionally taken into account for radiotherapy planning, as they provide valuable “real-time” functional information.^5^ The apparent diffusion coefficient (ADC) of a tumor lesion has been shown to function as a biomarker for prostate cancer on diagnostic scanners.^6^ MRL presents a novel opportunity to integrate ADC-values of a tumor lesion into daily plan adaptations and individualize radiotherapy.^7^ A prerequisite is the clinical translatability of ADC-measurements on MRL to a “gold standard” 3T diagnostic scanner (MRI_3T_).

In a previous study, it was demonstrated that ADC measurements of a region of interest in intraprostatic tumor lesions on MRL correlated with corresponding measurements on a diagnostic 3T MRI scanner (MRI_3T_).^8^ In that analysis, the MRIs on both scanners were performed prior to treatment initiation. However, as an initial step to evaluate, whether ADC measurements on an MRL might function as a biomarker enabling response assessment under RT, the longitudinal stability of ADC data gained on an MRL should be examined.

The purpose of this study is to longitudinally investigate the dynamics of lesion ADC in patients with prostate cancer undergoing MR-guided radiation therapy on an MR-Linac using the ADC values at a 3T MRI scanner as a reference standard.

## Patients and methods

### Participant sample, study design and MRI technique

All patients included in this prospective study were recruited in the M-base Pro 1.0^9^ or M-base HyPro 2.0 at our institution (ClinicalTrials.gov Identifiers: NCT02724670; NCT03880851). The study was conducted according to the guidelines of the Declaration of Helsinki, and approved by the Institutional Review Board of the medical faculty of Tuebingen University (No. 022/2016BO1, 14.03.2016 and No. 920/2018BO1, 10.07.2019). Informed consent was obtained from all subjects involved in the study. All patients consented to prospectively undergo multiple MRIs on an MRL and additionally on a MRI_3T_ at several points prior to and during RT. The aforementioned studies each examine a novel MR-adaptive concept for radiotherapy of primary localized prostate cancer. Between February 2019 and October 2021, 9 patients with biopsy-confirmed prostate cancer and available MRL and MRI_3T_ data sets prior to RT and under RT were included. All patients were treated daily on a 1.5T MRL (Elekta Unity^TM^, Philips, Stockholm, Sweden).^10^ All patients were treated according to national guidelines with either 39 × 2 Gy per fraction over eight weeks (M-base 1.0 study, n = 3 patients) or 20 × 3 Gy per fraction over four weeks (M-base Hypro 2.0 study, n = 6 patients) and additional neoadjuvant androgen deprivation therapy (ADT) of six months for intermediate risk patients and 24–36 months for high risk patients.^11^ The time point of the MRL and MRI_3T_ was during week 2 of RT in both treatment protocols. MRI technique, specifications and acquisition parameters of the examinations on both systems have been previously described.^8^ Most study participants in this study were used in the prior publication^8^, but only examinations prior to RT were analyzed. No lesion ADC dynamics during RT were reported in the previous study.^8^

### Lesion ADC evaluation

The ADC maps for MRL and MRI_3T_ for each patient, prior to and during radiotherapy, were independently presented to two readers (reader 1, a board certified radiation oncologist with 8 years of experience reader 2, a radiology resident with 4 years of experience). Both readers placed an elliptic region-of-interest (ROI) within the lesions for each patient in MRL and MRI_3T_ image sets. A dedicated workstation (GE Healthcare Centricity™ PACS RA1000, Milwaukee WI, USA) was utilized for image analysis using a dedicated software (syngo.via, Siemens Healthcare, Erlangen, Germany).

### Statistical analysis

Continuous variables were reported as mean and standard deviation. Paired t-tests were used for pair-wise pre- *vs*. during-treatment comparisons, as well as MRL *vs*. MRI-3T. Intraclass correlation coefficient (ICC, two-way, absolute agreement) was used to compute inter-reader agreement. An ICC of less than 0.4 signalizes poor agreement, of 0.40 to 0.59 indicates fair agreement, of 0.60 to 0.74 good agreement, and an ICC of 0.75 to 1.00 signalizes excellent agreement.^12^ Pearson Correlation coefficient was used to compare lesion ADC between MRL and MRI_3T_. Level of significance was set at 0.05. Statistical analyses were performed using SPSS (v26.0, IBM-Corp, Armonk, NY, USA).

## Results

**FIGURE 1.**
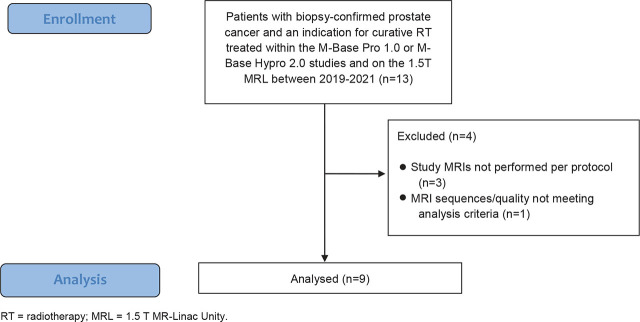
Flow diagram illustrating the inclusion/exclusion process.

A total of nine patients were included. Figure 1 delineates the the inclusion/exclusion process.

Table 1 summarizes patients’ characteristics. For each patient, the two imaging examinations prior to and during RT were successfully performed and evaluated.

The mean elapsed time between the baseline MRL exam and the MRL exam during radiotherapy was 26 days ± 14 days. The mean time difference between MRI_3T_ and MRL examinations at baseline was 1.7 days ± 1.7 days. Similarly, the mean time difference between MRI_3T_ and MRL examinations during radiotherapy was was 1.7 days ± 1.3 days.

Inter-reader reliability regarding lesion ADC measurement was excellent with ICC > 0.90 both at baseline and during radiotherapy (ICC for MRL at baseline was 0.927 and during radiotherapy was 0.976; ICC for MRI_3T_ at baseline was 0.978 and during radiotherapy was 0.998).

**FIGURE 2.**
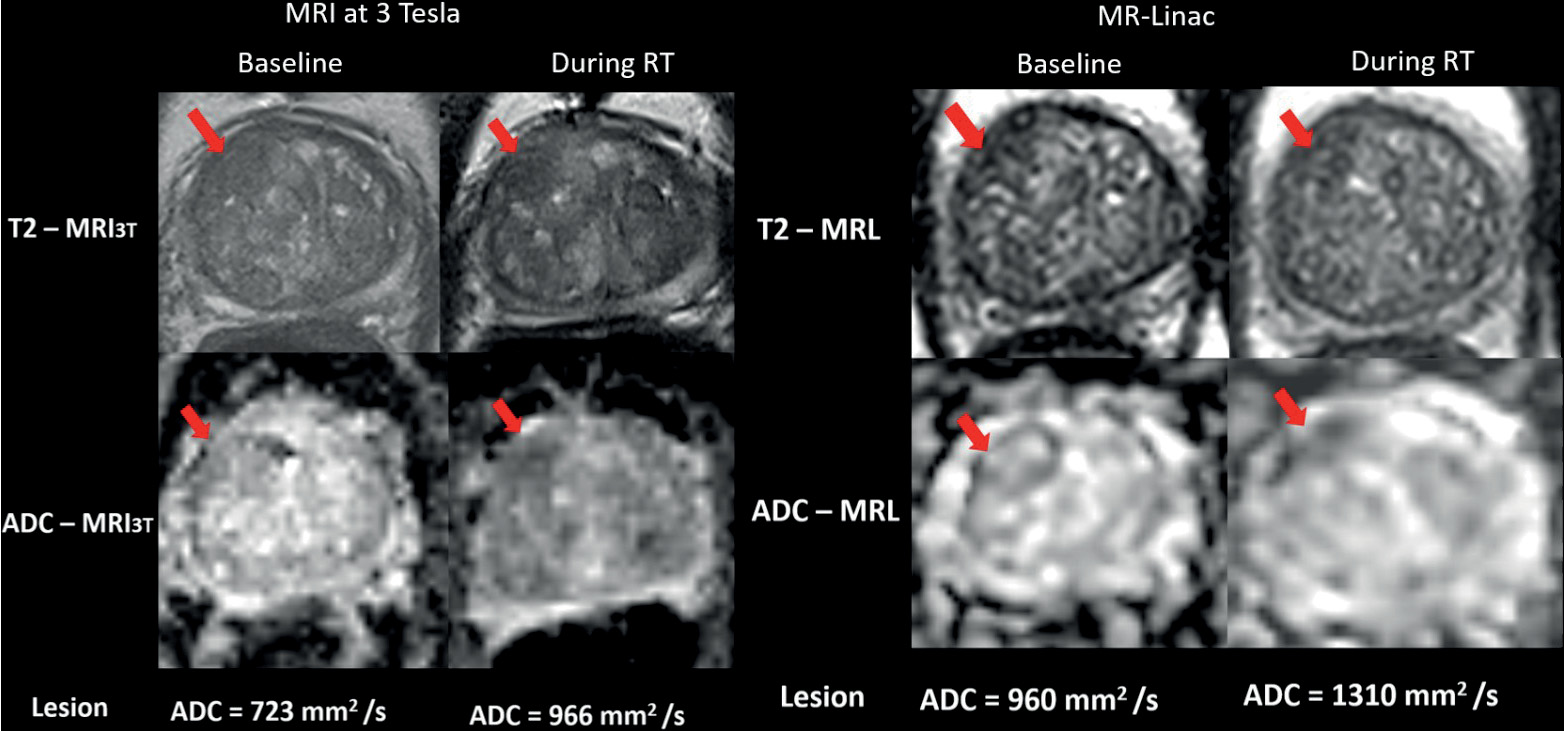
T2-weighted MR images (scan time: 2 minutes) and apparent diffusion coefficient (ADC) maps of a 66-year-old male patient with prostate cancer in the antero-apical region of the transition zone (red arrows) at baseline (left column) and during radiotherapy (right column) as recorded on MR-Linac (MRL) and on standard MRI at 3T (MRI3T). The figure shows similar dynamics of lesion ADC elevation during radiotherapy.

For reader 1, in both systems, there was a statistically significant elevation of lesion ADC during radiotherapy (Figure 2). Mean MRL-ADC at baseline was 0.97 ± 0.18 mm^2^/s *vs*. mean MRL-ADC during radiotherapy 1.38 ± 0.3 mm^2^/s, yielding a mean lesion ADC elevation of 0.41 ± 0.20 mm^2^/s, p <0.001. Mean MRI_3T_-ADC at baseline was 0.78 ± 0.165 mm^2^/s *vs*. mean MRI_3T_-ADC during radiotherapy 0.99 ± 0.175 mm^2^/s, yielding a mean lesion ADC elevation of 0.21 ± 0.96 mm^2^/s p <0.001.

**FIGURE 3.**
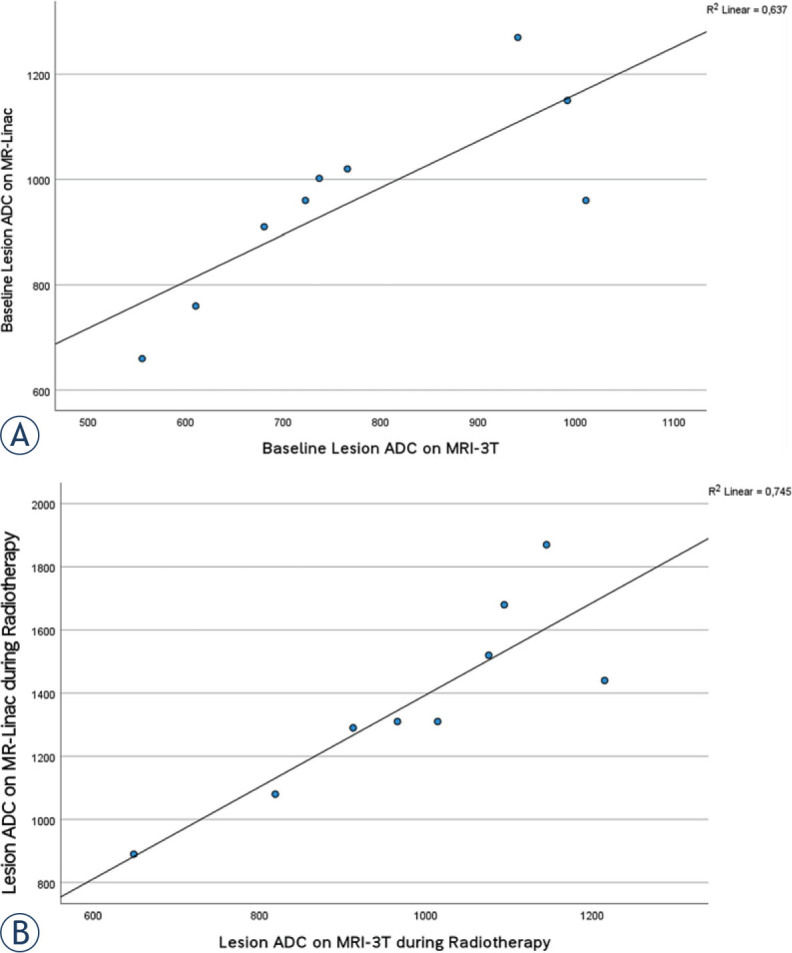
Scatter plots with a fitted line for ADC values as recorded on MR-Linac and MRI_3T_ for reader 1. **(A)** at baseline, **(B)** during radiotherapy (week 2). These plots illustrate the strong positive correlation between MRL-ADC and MRI_3T_-ADC at both time points.

The ADC values at MRL were consistently significantly higher than MRI_3T_ at baseline and during radiotherapy (p < 0.01). However, there was a strong positive correlation between MRL-ADC and MRI_3T_-ADC at baseline (r = 0.798, p = 0.01) and during radiotherapy (r = 0.863, p = 0.003) (Figure 3).

Similarly, for reader 2, in both systems, there was a statistically significant elevation of lesion ADC during radiotherapy (mean MRL-ADC at baseline was 1.0 ± 0.23 × 10^−3^ mm^2^/s *vs*. mean MRLADC during radiotherapy 1.36 ± 0.30 × 10^−3^ mm^2^/s, yielding a mean lesion ADC elevation of 0.36 ± 0.17 × 10^−3^ mm^2^/s, p <0.001). Mean MRI_3T_-ADC at baseline was 0.78 ± 0.17 × 10^−3^ mm^2^/s *vs*. mean MRI_3T_ADC during radiotherapy 1.0 ± 0.183 × 10^−3^ mm^2^/s, yielding a mean lesion ADC elevation of 0.22 ± 0.129 × 10^−3^ mm^2^/s p <0.001).

The ADC values at MRL were consistently significantly higher than MRI_3T_ at baseline and during radiotherapy (p < 0.001). However, there was a strong positive correlation between MRL-ADC and MRI_3T_-ADC at baseline (r = 0.872, p = 0.002) and during radiotherapy (r = 0.788, p = 0.012).

## Discussion

This prospective study compared lesion ADC values in patients with prostate carcinoma undergoing MR-guided radiotherapy on an MRL at 1.5 T to a diagnostic scanner at 3T. Absolute values of lesion ADC measurements differed while dynamics in the context of radiation therapy were comparable between the scanners. In both systems, there was a statistically significant elevation of lesion ADC during radiotherapy with a strong positive correlation of lesion ADC between the scanners.

### ADC changes during radiotherapy

ADC changes of the intraprostatic tumor are to be expected both during radiotherapy and during ADT and the correlation between ADC and prostate cancer aggressiveness has been shown before on diagnostic scanners.^17,18^ The mean ADC-values calculated in this study on both scanners are similar to values found in the literature: Tamada *et al.* reported mean ADC values of (untreated) tumor regions of 1.02 ± 0.25 × 10^−3^ mm^2^/s for the peripheral zone and of 0.94 ± 0.21 × 10^−3^ mm^2^/s for the transitional zone of the prostate.^13^ Van Schie *et al.* reported median ADC values in the tumor scanned on a diagnostic scanner of 1.08 ± 0.39 × 10^−3^ mm^2^/s (mean ± SD) prior to treatment and assessed changes of ADC prior to a hypofractionated RT and then weekly during RT in 73 patients in a similar manner as performed in our study. The group found a (non-significant) median increase of the ADC-value in the tumor of 7% for patients with concurrent ADT and a median increase of 20% for patients without ADT.^14^

### ADC as biomarker for response assessment and role of MRL

Moreover, ADC values and -changes have been shown to function as biomarkers for RT response in prostate cancer patients.^6,15^ Radiomics approaches seem promising in assessing response to RT, as performed by Abdollahi *et al*. prior to *vs*. after RT.^16^ In all of these studies and in prostate cancer diagnostics, a 3 T MRI scanner has been established as the gold standard for mpMRI.^17^ MRI_3T_ leads to optimal diagnostic images, often aided by suppression of peristalsis via intravenous application of butyl scopolamine or other agents. In contrast, on a 1.5 T MRL, the utilized sequences are optimized for fast and geometrically accurate image acquisition in an online workflow without routine administration of peristalisis suppressing medications or contrast agents. These possible limitations, in addition to technical differences of the hybrid system to diagnostic scanners^18^, pose the question whether an MRL can deliver comparable functional information during RT of prostate cancer.

In principle, the hybrid system offers fertile ground for further plan adaption in prostate carcinoma patients based on mpMRT findings such as ADC values since it offers daily MR-guided plan adaptations. Treatment individualization and plan adaptation under RT are a focus of research in other tumor entities as well.^7,19,20^ Longitudinal diffusion MRI on a 0.35 T hybrid system was already performed in small series for several other tumor entities with promising results (Yang *et al*.: three head and neck cancer patients and three sarcoma patients^21^; Shaverdian *et al.*: three rectal cancer patients^22^). On the 1.5 T MRL, Lawrence *et al*. report a high ADC repeatability and comparability to a diagnostic 1.5 T scanner for 59 patients with central nervous system tumors.^23^ Habrich *et al*. used a test-retest approach on 11 patients with head and cancers and showed to a high repeatability of ADC measurements on the 1.5 T MRL.^24^ In the context of prostate carcinoma, Habrich *et al*. also examined intravoxel incoherent motion (IVIM) and dynamic contrast enhanced (DCE) MRI changes over the course of a moderately hypofractionated RT in 20 patients, also indicating that longitudinal measurements of functional imaging parameters is feasible and could be used for response assessment in the future.^25^

However, concerning intraprostatic tumor lesions, the verification of longitudinal stability of ADC measurements on an MRL as performed in this study in a comparison to a latest generation 3 T diagnostic scanner has to the best of our knowledge not been performed yet.

In a previous study, we tested the clinical applicability based on qualitative and quantitative parameters of prostate MR images on an MRL against a MRI_3T_ at one point of time prior to starting RT. We were able to show a promising and comparable result of T2 weighted image quality, and lesion conspicuity and we reported comparable lesion ADC measurements between MRL and MRI_3T_.^8^

With the current work, we demonstrate longitudinal comparability and reliability between the two system during RT. This represents a necessary basis for future analyses of lesion changes over time on the MRL and confirms its potential for individualized treatment adaptations such as dose painting^26^ and response assessment during treatment.

This study has limitations. Firstly, the small sample size of patients who underwent multiple prostate imaging at both devices and the fact that only one time-point during radiotherapy was used for analysis. Multiple time-points during the course of radiotherapy should be analyzed to further validate the stability and comparability of ADC measurements. However, logistic challenges hindered further validation with an MRI_3T_ at more than one time point. Secondly, treatment regimens differed in this population (either 20 × 3 Gy oder 39 × 2 Gy, additional neoadjuvant ADT in 3 patients). Thirdly, the DWI acquisition parameters did not fully conform to the published recommendations of the MR-linac consortium, which were published after we had already included the patients in our study and predefinded the technical aspects of the utilized sequences.^27^

Nonetheless, this study depticts the reality and the challenges of clinical routine and its preliminary findings could be considered novel. Indeed, further prospective studies examining mpMRI data under RT and correlating those with clinical endpoints are desirable to advance individualized radiation treatment.

In conclusion, lesion ADC as measured on MRL increased significantly during radiotherapy and lesion ADC measurements on both systems showed similar dynamics. These preliminary findings are promising but need large-scale validation. Once validated, lesion ADC on MRL might be used as a biomarker for real-time assessment of tumor response in patients with prostate cancer undergoing MR-guided radiation therapy.
